# Patient Experience at US Hospitals Following the Caregiver Advise, Record, Enable (CARE) Act

**DOI:** 10.1001/jamanetworkopen.2023.11253

**Published:** 2023-05-01

**Authors:** Courtney R. Lee, Elizabeth Taggert, Norma B. Coe, Paula Chatterjee

**Affiliations:** 1 Leonard Davis Institute of Health Economics, University of Pennsylvania, Philadelphia; 2 Department of Medicine, Division of General Internal Medicine, University of Pennsylvania Perelman School of Medicine, Philadelphia; 3 Department of Medical Ethics and Health Policy, University of Pennsylvania, Philadelphia

## Abstract

**Importance:**

Communication with caregivers is often not established or standardized during hospitalization. The Caregiver Advise, Record, Enable (CARE) Act is a state-level policy designed to facilitate communication among patients, caregivers, and clinical care teams during hospitalization to improve patient experience; 42 states have passed this policy since 2014, but whether it was associated with achieving these goals remains unknown.

**Objective:**

To determine whether passage of the CARE Act was associated with improvements in patient experience.

**Design, Setting, and Participants:**

This cohort study used a difference-in-differences analysis of short-term, acute-care US hospitals from 2013 to 2019 to analyze changes in patient experience before vs after CARE Act implementation in hospitals located in states that passed the CARE Act compared with those in states that did not. Analyses were performed between September 1, 2021, and July 31, 2022.

**Exposure:**

Time-varying indicators for whether a hospital was in a state that passed the CARE Act.

**Main Outcomes and Measures:**

Patient-reported experience via the Hospital Consumer Assessment of Healthcare Providers and Systems survey.

**Results:**

A total of 2763 hospitals were included, with 2188 hospitals in CARE Act states and 575 in non–CARE Act states. There were differential improvements in patient experience in the measures of communication with nurses (unadjusted mean [SD] score, 78.40% [0.42%]; difference, 0.18 percentage points; 95% CI, 0.07-0.29 percentage points; *P* = .002), communication with physicians (mean [SD] score, 80.00% [0.19%]; difference, 0.17 percentage points; 95% CI, 0.06-0.28 percentage points; *P* = .002), and receipt of discharge information (mean [SD] score, 86.40% [0.22%]; difference, 0.11 percentage points; 95% CI, 0.02-0.21 percentage points; *P* = .02) among CARE Act states compared with non–CARE Act states after policy passage. In subgroup analyses, improvements were larger among hospitals with lower baseline Hospital Consumer Assessment of Healthcare Providers and Systems performance on measures of communication with nurses, communication with physicians, and overall hospital rating.

**Conclusions and Relevance:**

These findings suggest that implementation of the CARE Act was associated with improvements in several measures of patient experience. Policies that formally incorporate caregivers into patient care during hospitalization may improve patient outcomes.

## Key Points

**Question:**

Was passage of the Caregiver Advise, Record, Enable (CARE) Act, a state-level policy designed to facilitate communication among patients, caregivers, and clinical care teams during hospitalization, associated with differential improvements in patient experience?

**Findings:**

In this cohort study of 2763 short-term, acute-care US hospitals from 2013 to 2019, differential improvements in patient experience were found across multiple measures, including communication with nurses and physicians and receipt of discharge information, among CARE Act states compared with non–CARE Act states after policy passage.

**Meaning:**

These findings suggest that policies that formally integrate communication may improve patient outcomes.

## Teaser

This cohort study examines whether passage of the Caregiver Advise, Record, Enable (CARE) Act was associated with improvements in patient experience.

## Introduction

Transitioning from the hospital to a home or community setting represents a vulnerable period for patients and caregivers. Despite the growth of discharge planning services,^1,2^ patients and caregivers still describe feeling ill-prepared for or sidelined from the discharge planning process.^3,4^ As hospital length of stay has shortened, caregivers have become increasingly responsible for postdischarge care tasks, often without receiving formal training, education, or integration into the plan of care.^5^ Failure to involve caregivers may adversely affect the patient’s experience of care during hospitalization, as well as the quality of care provided in the postdischarge setting. These consequences might be particularly important for the 20% of older adults who are readmitted to the hospital within 30 days of discharge.^6^

Policies designed to standardize communication among clinical care teams, patients, and caregivers may play an important role in improving patient experience and shaping patient outcomes.^7^ The Caregiver Advise, Record, Enable (CARE) Act is one such state-level policy that requires hospitals to advise patients of the opportunity to designate a caregiver, record their name and contact information in the health record, and enable the caregiver by consulting and providing education on medical or nursing tasks to be performed at home.^8,9^ Since 2014, 42 states have passed the CARE Act with the goal of supporting patients and caregivers during care transitions. One of the primary goals of the policy was to improve patient experience with care by standardizing communication among patients, caregivers, and clinical care teams.^9,10^ Smaller hospital-based interventions that integrate caregivers into discharge planning have shown success across similar outcomes,^1,11,12^ but whether the CARE Act has achieved its stated goals for patients remains unknown.

Understanding state-level policies that standardize inclusion of caregivers in health care delivery can inform how hospitals engage with patients and caregivers in ways that can improve patient outcomes. Here, we used a difference-in-differences approach with an event study specification to compare changes in patient experience among hospitals in states that passed the CARE Act vs hospitals in states that did not.

## Methods

### Data Sources

In this national cohort study, we identified US hospitals using the American Hospital Association’s Annual Survey from 2013 to 2019. This survey includes hospital characteristics such as teaching status, ownership, number of hospital beds, geographic region, number of Medicare inpatient days, number of Medicaid inpatient days, and total inpatient facility days. We performed a sequence of exclusions to limit the sample to acute-care, short-term hospitals (eFigure 1 in Supplement 1). We used the Centers for Medicare & Medicaid Services (CMS) Provider of Services file to exclude hospitals that did not have consistent CMS certification numbers in every year of the study or that had an indicator notifying CMS of termination of services. This was because changes in CMS certification numbers or indicators of termination can be associated with mergers or closures that may reflect differences in underlying hospital quality. We also excluded hospitals in US territories (American Samoa, Guam, Northern Mariana Islands, US Virgin Islands, and Puerto Rico).

We obtained the exposure, whether a state enacted the CARE Act in a given year, from the American Association for Retired Persons Public Policy Institute (eTable 1 and eTable 2 in Supplement 1).^9^ To evaluate patient experience during hospitalization, we obtained hospital-year performance on the Hospital Consumer Assessment of Healthcare Providers and Systems (HCAHPS) Survey from CMS’s Hospital Compare database. This survey is administered to a random sample of eligible patients between 2 and 42 days after hospital discharge. Eligible patients were at least 18 years old at the time of admission, had at least 1 overnight stay with a nonpsychiatric primary diagnosis, and were discharged alive. Surveys are administered through mail, email, or telephone. Results are publicly reported and lag by 1 year (eg, data reported in 2016 reflect survey results from 2015).^13,14^ We assigned data to capture the year in which the survey data were collected (eTable 3 in Supplement 1). Finally, we collected annual county-level proportions of the population aged 65 years and older from the American Community Survey, and annual hospital case mix indices and urbanicity from the CMS Impact files.

Because the study used deidentified, hospital-level data, it was exempt from institutional review board approval and the need for informed consent, as authorized by 45 CFR 46.104, category 4. The report follows the Strengthening the Reporting of Observational Studies in Epidemiology (STROBE) reporting guidelines.

### Exposure Variable

We created a time-varying indicator for each state’s CARE Act passage status. Among states that never passed the CARE Act during the study period (January 2013 to December 2019), this variable was equal to 0 in all years. In states that passed the CARE Act, this variable equaled 0 in years before passage and 1 in the year after passage and onward (eg, if passage was in 2016, then the indicator variable was 0 before 2017, and 1 in 2017 and onward) (eTable 1 and eTable 2 in Supplement 1). This 1-year lag was to allow time for policy implementation.^13,14^ Although the CARE Act is a state-level policy, several of its provisions are uniform across states (eTable 4 in Supplement 1).

### Primary Outcomes

In accordance with the stated goals of the CARE Act,^9^ the primary outcome was patient-reported experience. Patient experience was measured using the HCAHPS survey at the hospital-year level. HCAHPS includes a series of questions on communication with clinical care teams and overall hospital experience (eFigure 2 in Supplement 1). Communication was captured across 5 measures: communication with nurses (percentage of patients who reported that nurses always communicated well), communication with physicians (always communicated well), communication about medications (always explained before they were given), discharge information (yes to receiving information about what to do in their recovery at home), and care transition information (strongly agree that they understood their care when they left the hospital). Overall hospital experience was evaluated across 2 measures: overall hospital rating (percentage of patients who assigned a score of 9 or 10 on 10-point scale) and whether the patient would recommend the hospital to others (percentage who responded definitely yes to whether they would recommend the hospital). We excluded hospitals that did not have complete data for all the outcomes in each year of the study period (eFigure 3 in Supplement 1).

### Statistical Analysis

The analysis was conducted using a balanced panel of hospital-year observations from 2013 to 2019. We compared characteristics between hospital-year observations in states that passed the CARE Act vs those that did not. We then assessed the parallel trends assumption using visual plots of each outcome over time, by dividing hospitals within states into 3 groups according to the median year of CARE Act passage across states (2016). These groups were specified as early adopters (ie, passed the CARE Act before 2016), late adopters (ie, passed the CARE Act after 2016), and nonadopters (ie, did not pass the CARE Act). We also formally modeled preexposure hospital-year observations using an interaction term between the exposure (yes or no for whether a hospital was in a CARE Act state) and the number of years before exposure to assign statistical significance to whether preperiod trends in the outcomes were similar in CARE Act vs non–CARE Act states.

To evaluate whether the CARE Act was associated with improvements in patient experience, we used a difference-in-differences approach with an event study specification developed by Callaway et al^15^ (eAppendix in Supplement 1). We chose this specification because it allowed us to disaggregate group-time average treatment effects, which were relevant to the staggered timing of CARE Act passage across states (eTable 2 in Supplement 1). We estimated differential changes in outcomes by comparing hospitals in CARE Act states with hospitals in states that had yet to pass the CARE Act or never passed the CARE Act. We adjusted for time-varying hospital (number of beds and ownership type) and county (proportion of residents aged ≥65 years) characteristics. We also adjusted for state and year fixed effects, and clustered SEs at the state to reflect the level of the policy exposure.^16^

We performed 1 subgroup analysis. Because prior work has shown that hospitals with low baseline performance have greater improvements in HCAHPS over time,^17^ we hypothesized that hospitals with lower baseline performance might demonstrate larger differential improvements after the CARE Act. We used data from 2013 to standardize baseline performance across all HCAHPS measures into a *z* score. Then we divided hospitals into quartiles on the basis of their scores: hospitals in the bottom 3 quartiles were considered low performing, whereas hospitals in the top quartile were high performing. All subgroup analyses were considered exploratory in nature.

We conducted several sensitivity analyses. First, to evaluate whether other state-level policy changes during the study period (eg, Medicaid expansion) contributed to the observed association, we repeated analyses including an interaction term between state and year. Second, we repeated analyses using a less-stringent definition of high-quality patient experience by combining top-box (as used in main analyses) and middle-box (categorized as usually, agree, or sometimes for communication measures, 7 or 8 for overall hospital rating, and probably yes for recommending the hospital to others) responses into a composite for each HCAHPS measure.

We used SAS statistical software version 9.4 (SAS Institute) to construct the data set and Stata statistical software version 16.1 (StataCorp, LLC) to conduct statistical analyses. We used a 2-sided *P* < .05 as the significance threshold. Analyses were performed between September 1, 2021, and July 31, 2022.

## Results

**Figure 1.**
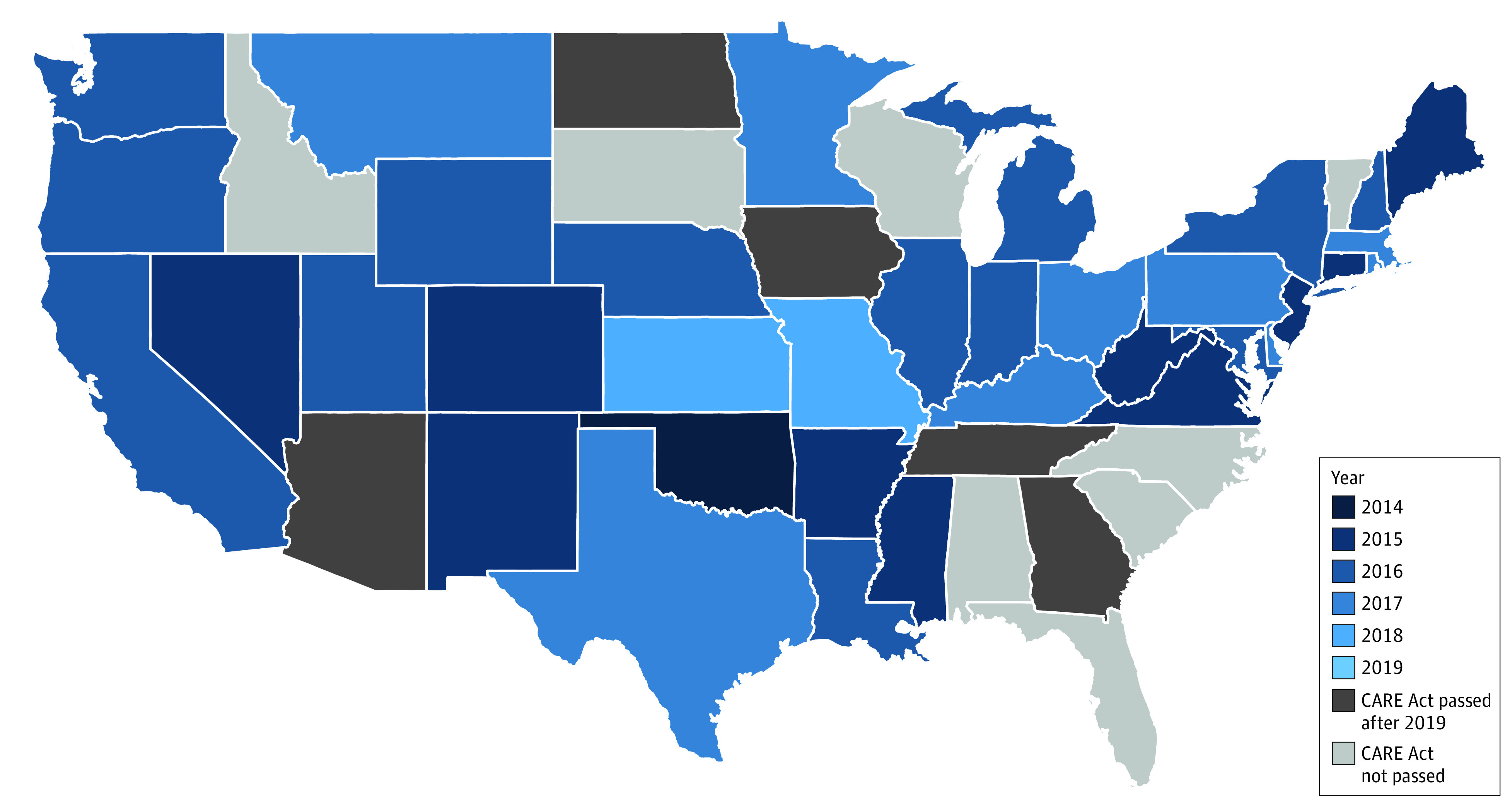
State-Level Adoption of the Caregiver Advise Enable Record (CARE) Act by Year Of the 50 states comprising the study sample, 38 exposed states passed the CARE Act at varying time periods during the study period (2013-2019). These states were defined as exposed in the following year after passage and onward. The unexposed states include 5 states (Tennessee, North Dakota, Iowa, Georgia, and Arizona) that passed the CARE Act after the study period and 7 states (Alabama, Florida, Idaho, North Carolina, South Carolina, South Dakota, Vermont, and Wisconsin) that have not passed the CARE Act.

From 2013 to 2019, 37 states passed the CARE Act (Figure 1). After 2019, 5 additional states passed the Act but were considered unexposed in this analysis (eTable 1 and eTable 2 in Supplement 1).

Among 3019 short-term, acute-care hospitals, we excluded 256 (8.5%) with missing data for HCAHPS in at least 1 hospital-year during the study period (eFigure 3 in Supplement 1). Hospitals in CARE Act states were less likely to demonstrate missingness (468 hospital-years [3.0%]) compared with hospitals in non–CARE Act states (422 hospital-years [7.7%]). Missing observations were more likely to come from small hospitals (501 hospital-years [56.3%]) and for-profit hospitals (338 hospital-years [60.5%]) (eFigure 3 in Supplement 1). The final sample included 2763 hospitals, of which 2188 were in CARE Act states and 575 in non–CARE Act states (eTable 5 and eTable 6 in Supplement 1).

Hospital-year observations in CARE Act states were more often from nonprofit hospitals (10 683 hospitals [69.8%] vs 2194 hospitals [54.5%]) and were less often located in the South (4830 hospitals [31.5%] vs 3108 hospitals [77.2%]) compared with non–CARE Act states (eTable 6 in Supplement 1). Visual plots of outcomes over time and formal regression models of preexposure observations did not suggest violations of the parallel trends assumptions (eFigure 5, eFigure 6, and eTable 7 in Supplement 1).

**Figure 2.**
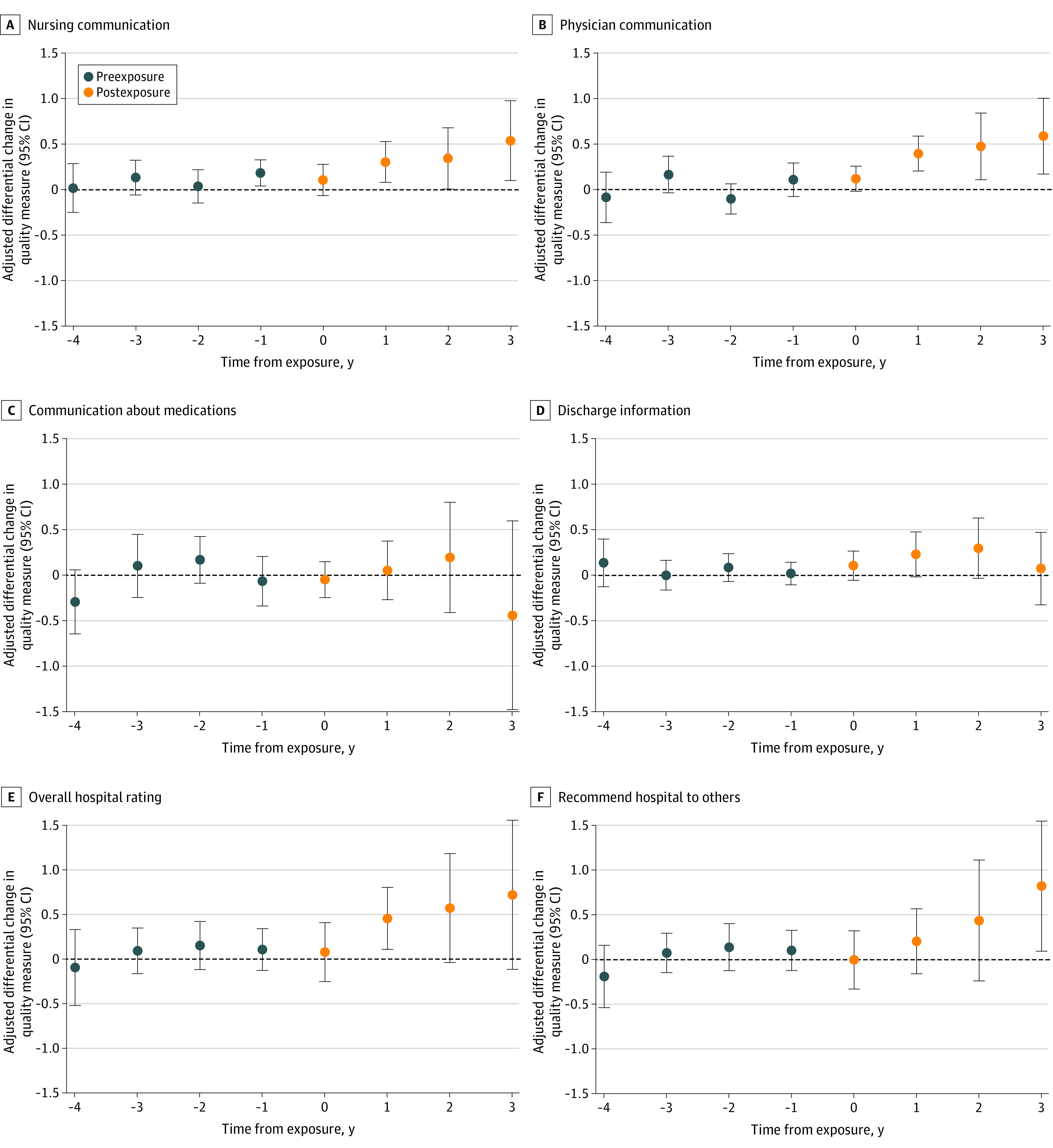
Estimates of Adjusted Differences in Patient Experience Between Hospitals in Caregiver Advise Enable Record (CARE) Act States vs Non–CARE Act States The absolute adjusted differences in quality measures between hospitals located in exposed states and not yet exposed states for each outcome are plotted. The x-axis represents the number of years relative to CARE Act passage with event years greater than 4 years before exposure and 3 years after exposure excluded from the analyses. The sample consisted of 19 383 hospital-year observations and represented 38 exposed and 13 unexposed states. All models were adjusted for hospital characteristics (number of beds and ownership type), county-level proportion of residents aged 65 years and older, and year and state fixed effects to reflect the level of the policy exposure.

There were significant differential improvements across several measures of patient experience. Hospitals in CARE Act states had a significant differential improvement in the quality of nursing communication compared with non–CARE Act states after passage of the Act (unadjusted mean [SD] score, 78.40% [0.42%]; difference, 0.18 percentage points; 95% CI, 0.07-0.29 percentage points; *P* = .002) (Table 1 and Figure 2). There were also significant differential improvements of similar magnitude in physician communication (mean [SD] score, 80.00% [0.19%]; difference, 0.17 percentage points; 95% CI, 0.06-0.28 percentage points; *P* = .002) and discharge information (mean [SD] score, 86.40% [0.22%]; difference, 0.11 percentage points; 95% CI, 0.02-0.21 percentage points; *P* = .02) among hospitals in CARE Act states compared with non–CARE Act states. Finally, there were significant differential improvements in overall hospital rating (mean [SD] score, 70.10% [0.41%]; difference, 0.22 percentage points; 95% CI 0.05-0.39 percentage points; *P* = .01). Similar patterns of improvement were observed for whether patients would recommend the hospital to others, although they did not reach statistical significance.

**Table 1.** Differential Changes in Patient Experience Between Hospitals in CARE Act and Non–CARE Act States, 2013-2019

Patient experience quality domain	Unadjusted score, mean (SD), %	Adjusted differential change, percentage points (95% CI)^a^	*P* value
Nursing communication	78.40 (0.42)	0.18 (0.07 to 0.29)	.002
Doctor communication	80.00 (0.19)	0.17 (0.06 to 0.28)	.002
Communication on medications	63.20 (0.20)	0.02 (−0.15 to 0.19)	.81
Discharge information	86.40 (0.22)	0.11 (0.02 to 0.21)	.02
Care transition information	50.80 (0.30)	−0.05 (−0.17 to 0.08)	.45
Overall hospital rating	70.10 (0.41)	0.22 (0.05 to 0.39)	.01
Recommend hospital to others	70.10 (0.06)	0.15 (−0.04 to 0.34)	.12

Abbreviation: CARE, Caregiver Advise Enable Record.

^a^ Differential change represents the estimated percentage point difference between hospitals in CARE Act states compared with those in non–CARE Act states after adjusting for number of beds, ownership type, and proportion of residents aged 65 years and older. We also adjusted for state and year fixed effects, and clustered SEs at the state to reflect the level of the policy exposure.

Differential improvements continued in subsequent years after passage of the CARE Act for outcomes related to patient experience. Three years after CARE Act passage, hospitals in CARE Act states had larger improvements in nursing communication (difference, 0.54 percentage points; 95% CI, 0.10-0.98 percentage points; *P* = .02), physician communication (difference, 0.59 percentage points; 95% CI, 0.17-1.00 percentage points; *P* = .01), and whether a patient would recommend the hospital to others (difference, 0.82 percentage points; 95% CI, 0.10-1.55 percentage points; *P* = .03) compared with hospitals in non–CARE Act states (Figure 2 and eTable 8 in Supplement 1). We also found larger improvements in overall hospital rating in the years following the CARE Act, although they were not significant (Figure 2).

In subgroup analyses comparing low vs high baseline performing hospitals on HCAHPS, we found differential improvements of similar magnitude on nursing communication and physician communication (Table 2). However, differential improvements in overall hospital rating were larger among hospitals with low baseline performance (difference, 0.21 percentage points; 95% CI, 0.04 to 0.39 percentage points; *P* = .02) compared with those with high baseline performance (difference, 0.11 percentage points; 95% CI, −0.11 to 0.33 percentage points; *P* = .31). All findings related to subgroup analyses were interpreted as exploratory.

**Table 2.** Subgroup Analyses of Hospitals by Better vs Worse Baseline Performance on Patient Experience

Patient experience quality domain	Baseline HCAHPS performance					
	Worse (lower HCAHPS scores)			Better (higher HCAHPS scores)		
	Unadjusted score, mean (SD), %	Adjusted differential change, percentage points (95% CI)^a^	*P* value	Unadjusted score, mean (SD), %	Adjusted differential change, percentage points (95% CI)^a^	*P* value
Nursing communication	77.20 (0.53)	0.16 (0.05 to 0.27)	.004	82.10 (0.07)	0.16 (−0.03 to 0.34)	.09
Physician communication	78.90 (0.15)	0.16 (0.05 to 0.27)	.003	83.50 (0.33)	0.15 (−0.02 to 0.33)	.08
Communication on medications	61.90 (0.32)	−0.01 (−0.18 to 0.16)	.94	67.10 (0.19)	0.004 (−0.20 to 0.20)	.97
Discharge information	85.70 (0.30)	0.10 (0.01 to 0.20)	.03	88.40 (<0.01)	0.06 (−0.06 to 0.18)	.31
Care transition information	49.20 (0.41)	−0.08 (−0.20 to 0.05)	.23	55.70 (0.04)	−0.07 (−0.28 to 0.15)	.53
Overall hospital rating	68.00 (0.57)	0.22 (0.04 to 0.39)	.02	76.60 (0.10)	0.11 (−0.11 to 0.33)	.31
Recommend hospital to others	67.80 (0.07)	0.16 (−0.03 to 0.35)	.1	76.90 (0.46)	0.03 (−0.18 to 0.25)	.77

Abbreviation: HCAHPS, Hospital Consumer Assessment of Healthcare Providers and Systems.

^a^ Differential change represents the estimated percentage point difference between hospitals in Caregiver Advise Enable Record (CARE) Act states compared with those in non–CARE Act states after adjusting for number of beds, ownership type, and proportion of residents aged 65 years and older. We also adjusted for state and year fixed effects, and clustered SEs at the state to reflect the level of the policy exposure.

Findings were robust to sensitivity analyses. We found consistent results when we added an interaction term to account for other state-level policies passed during the study period (eTable 9 in Supplement 1). Substantive findings were also unchanged when we modeled outcomes with the inclusion of top-box and middle-box responses (eTable 10 in Supplement 1).

## Discussion

In this national cohort study, we found that passage of the CARE Act was associated with differential improvements in patient experience. Improvements in patient experience persisted with each year since passage of this policy and were larger among hospitals with low baseline performance. These findings suggest that collaborating with caregivers during discharge planning is associated with better patient experience and outcomes.

There are several potential explanations for these findings. First, the CARE Act requires hospitals to offer caregivers training and education in postdischarge medical and nursing tasks.^8^ Given that discharge planning is a multidisciplinary process,^18^ it is not surprising that the policy led to improvements in communication with nurses and physicians, who as frontline health care professionals, routinely notify caregivers and provide predischarge counseling.^14,19,20^ Second, patients’ perceptions of discharge preparedness is one of the factors most associated with satisfaction with care and also the most responsive to communication and education.^21^ As a result, better communication may have improved patients’ perceptions of discharge preparedness, in turn, leading patients to report a better experience of care.

Although our findings are consistent with prior work^22,23^ that has demonstrated better communication and counseling during discharge planning to be associated with higher patient satisfaction, our effect sizes are small compared with prior randomized clinical trials and difference-in-difference studies evaluating the effectiveness of various interventions to improve HCAHPS scores.^17,24^ However, the existing literature on HCAHPS-specific interventions is limited in terms of its methods and generalizability, given its focus on specific patient populations and the absence of caregiver-related interventions.^25^ Qualitative analyses of CARE Act implementation have found improved identification and documentation of caregivers in the health records, without similar modifications to clinicians’ interactions with caregivers.^14^ In addition, the lack of significant association between the CARE Act and the outcome pertaining to the quality of care transitions may be because provisions of the CARE Act addressed some, but not all, of the elements in the quality measure. This gap between documentation and integration may contribute to the small effect sizes observed in our analyses.

The benefits of the CARE Act may also motivate hospitals to rethink how they collaborate with caregivers and improve transitions of care for patients and their caregivers living in underserved communities.^26^ For example, caregiver involvement could be formalized across other domains of delivery, including emergency department visits or end-of-life care. Furthermore, as hospitals and health care systems consider best practices to advance health equity and address social determinants of health, those that take a systematic approach to integrating caregivers might be better able to assess patients’ structurally determined risks and offset negative health outcomes associated with caregiving, which disproportionately affect individuals from minoritized racial and ethnic backgrounds.^27,28^

The COVID-19 pandemic has exposed the unaddressed needs and important contributions of caregivers.^27,29^ In light of this, many states are using their home-based and community-based service funds from the federal government, which must be spent by the end of 2024, to support caregiver training or respite care.^30^ However, one challenge to implementing such policies is that many caregivers do not self-identify as such, which may limit their ability to access support services.^31,32^ As a result, recent state strategies have focused on increasing self-identification of caregivers and raising awareness of services and supports.^31^ The CARE Act provisions to recognize caregivers during hospitalization may offer another valuable opportunity for identification and outreach.

### Limitations

There are several limitations to this study. First, we focused on a narrow set of outcomes related to patient experience because they follow directly from the CARE Act’s goals. However, hospital quality and patient outcomes can be defined broadly across other domains that we did not examine. Second, we relied on hospital-level data and are unable to assess the fidelity of CARE Act implementation among clinical care teams, patients, and caregivers. Third, the average response rate for HCAHPS is below 40%,^33^ which may limit the generalizability of our findings. Nonetheless, HCAHPS scores are standardized, publicly reported, and used as a valid assessment of hospital quality in federal programs. Fourth, our study did not evaluate caregiver outcomes, such as the financial and nonfinancial costs of additional caregiver responsibilities assumed after the CARE Act. Fifth, despite our use of causal inference methods, we are unable to rule out unmeasured residual confounding that may have biased these findings. Hospital characteristics that differ within states and over time, such as varying hospital participation in value-based payment programs or hospital staff ratios, may not have been adequately controlled for in the current model. However, investigating the role of hospital characteristics in CARE Act implementation presents an opportunity for future study. Sixth, we observed missingness not at random, which may bias our findings. However, missing data represented less than 10% of our sample; thus, we expect the contributions of bias to be small.

## Conclusions

The findings of this study suggest that from 2013 to 2019, the passage of the CARE Act was associated with differential improvements in patient experience. These findings highlight the importance of involving patients and caregivers during care transitions and underscores the role of state policies in improving patient outcomes.

## Notes

Supplement 1. **eFigure 1.** Flow Diagram of Sample Creation **eTable 1.** State-Level Passage of the CARE Act and Year Assigned in Analyses **eTable 2.** Timeline of State-Level Adoption of the CARE Act by State **eTable 3.** Hospital Consumer Assessment of Healthcare Providers and Systems (HCAHPS) Measurement Dates and Year Assignments for Quality Measures **eTable 4.** Overview of CARE Act Policy by State **eTable 5.** State-Level Caregiving Initiatives by State **eFigure 2.** Hospital Consumer Assessment of Healthcare Providers and Systems (HCAHPS) Survey Instrument **eFigure 3.** Frequency of Missingness for Hospitals Over the Study Period (2013-2019) **eTable 6.** Hospital Characteristics by CARE Act Passage Across All Years (2013-2019) **eAppendix.** Estimation Method Used in Callaway and Sant’Anna Differences-in-Differences Approach **eTable 7. **Regression Estimates for Parallel Pre-trends **eFigure 5.** Performance on HCAHPS, Unadjusted Means Over Time—Hospitals in CARE Act States vs. Non-CARE Act States **eFigure 6.** Estimates of Adjusted Differences in Care Transition Information Between Hospitals in CARE Act States vs Non-CARE Act States **eTable 8.** Differential Changes in Patient Experience and Readmissions Scores Between Hospitals in CARE Act and Non-CARE Act States by Time From Exposure **eTable 9.** Regression Estimates After Adding Interaction Term Between State and Year **eTable 10.** Regression Estimates Using Composite Scores of Top Box and Middle Box Responses

Supplement 2.
Data Sharing Statement

## References

[1] Naylor MD, Brooten D, Campbell R, . Comprehensive discharge planning and home follow-up of hospitalized elders: a randomized clinical trial. JAMA. 1999;281(7):613-620. doi:10.1001/jama.281.7.613 10029122

[2] Gonçalves-Bradley DC, Lannin NA, Clemson L, Cameron ID, Shepperd S. Discharge planning from hospital. Cochrane Database Syst Rev. 2022;2(2):CD000313. doi:10.1002/14651858.CD000313.pub6 35199849 PMC8867723

[3] Reinhard S. Home alone revisited: family caregivers providing complex care. Innov Aging. 2019;3(suppl 1):S747-S748. doi:10.1093/geroni/igz038.2740

[4] Shyu YIL. The needs of family caregivers of frail elders during the transition from hospital to home: a Taiwanese sample. J Adv Nurs. 2000;32(3):619-625. doi:10.1046/j.1365-2648.2000.01519.x 11012804

[5] Carlin C, David G. Reduced health care utilization among elderly patients with informal caregivers. Perm J. 2019;23:18-173. doi:10.7812/TPP/18-173 31314726 PMC6636532

[6] Jencks SF, Williams MV, Coleman EA. Rehospitalizations among patients in the Medicare fee-for-service program. N Engl J Med. 2009;360(14):1418-1428. doi:10.1056/NEJMsa0803563 19339721

[7] Blair J, Volpe M, Aggarwal B. Challenges, needs, and experiences of recently hospitalized cardiac patients and their informal caregivers. J Cardiovasc Nurs. 2014;29(1):29-37. doi:10.1097/JCN.0b013e3182784123 23416934 PMC3726572

[8] Reinhard S, Ryan E. Stepping up to support family caregivers. AARP blogs: thinking policy. June 7, 2016. Accessed July 8, 2022. https://blog.aarp.org/thinking-policy/stepping-up-to-support-family-caregivers

[9] Reinhard SC, Young HM, Ryan E, Choula RB. The CARE Act Implementation: Progress and Promise. AARP Public Policy Institute; 2019.

[10] Dawson WD, Bangerter LR, Splaine M. The politics of caregiving: taking stock of state-level policies to support family caregivers. Public Policy Aging Rep. 2020;30(2):62-66. doi:10.1093/ppar/praa005

[11] Rodakowski J, Rocco PB, Ortiz M, . Caregiver integration during discharge planning for older adults to reduce resource use: a metaanalysis. J Am Geriatr Soc. 2017;65(8):1748-1755. doi:10.1111/jgs.14873 28369687 PMC5555776

[12] Shyu YIL, Liang J, Wu CC, . A pilot investigation of the short-term effects of an interdisciplinary intervention program on elderly patients with hip fracture in Taiwan. J Am Geriatr Soc. 2005;53(5):811-818. doi:10.1111/j.1532-5415.2005.53253.x 15877556

[13] Rodakowski J, Leighton C, Martsolf GR, James AE. Caring for family caregivers: perceptions of CARE Act compliance and implementation. Qual Manag Health Care. 2021;30(1):1-5. doi:10.1097/QMH.0000000000000278 33229997 PMC7844420

[14] Leighton C, Fields B, Rodakowski JL, . A multisite case study of Caregiver Advise, Record, Enable Act implementation. Gerontologist. 2020;60(4):776-786. doi:10.1093/geront/gnz011 30726908

[15] Callaway B, Sant’Anna PHC. Difference-in-differences with multiple time periods. J Econom. 2021;225(2):200-230. doi:10.1016/j.jeconom.2020.12.001

[16] Bertrand M, Duflo E, Mullainathan S. How much should we trust differences-in-differences estimates? Q J Econom. 2004;119(1):249-275. doi:10.1162/003355304772839588

[17] Mann RK, Siddiqui Z, Kurbanova N, Qayyum R. Effect of HCAHPS reporting on patient satisfaction with physician communication. J Hosp Med. 2016;11(2):105-110. doi:10.1002/jhm.2490 26404621

[18] Kripalani S, Jackson AT, Schnipper JL, Coleman EA. Promoting effective transitions of care at hospital discharge: a review of key issues for hospitalists. J Hosp Med. 2007;2(5):314-323. doi:10.1002/jhm.228 17935242

[19] Wong EL, Yam CH, Cheung AW, . Barriers to effective discharge planning: a qualitative study investigating the perspectives of frontline healthcare professionals. BMC Health Serv Res. 2011;11(1):242. doi:10.1186/1472-6963-11-242 21955544 PMC3190337

[20] Samuels-Kalow ME, Stack AM, Porter SC. Effective discharge communication in the emergency department. Ann Emerg Med. 2012;60(2):152-159. doi:10.1016/j.annemergmed.2011.10.023 22221840

[21] Bull MJ, Hansen HE, Gross CR. Predictors of elder and family caregiver satisfaction with discharge planning. J Cardiovasc Nurs. 2000;14(3):76-87. doi:10.1097/00005082-200004000-00010 10756476

[22] Lindpaintner LS, Gasser JT, Schramm MS, Cina-Tschumi B, Müller B, Beer JH. Discharge intervention pilot improves satisfaction for patients and professionals. Eur J Intern Med. 2013;24(8):756-762. doi:10.1016/j.ejim.2013.08.703 24075842

[23] Coleman EA, Roman SP, Hall KA, Min SJ. Enhancing the care transitions intervention protocol to better address the needs of family caregivers. J Healthc Qual. 2015;37(1):2-11. doi:10.1097/01.JHQ.0000460118.60567.fe 26042372

[24] Harper CM, Dong Y, Thornhill TS, . Can therapy dogs improve pain and satisfaction after total joint arthroplasty? a randomized controlled trial. Clin Orthop Relat Res. 2015;473(1):372-379. doi:10.1007/s11999-014-3931-0 25201095 PMC4390934

[25] Davidson KW, Shaffer J, Ye S, . Interventions to improve hospital patient satisfaction with healthcare providers and systems: a systematic review. BMJ Qual Saf. 2017;26(7):596-606. doi:10.1136/bmjqs-2015-004758 27488124 PMC5290224

[26] Griffin JM, Kaufman BG, Bangerter L, . Improving transitions in care for patients and family caregivers living in rural and underserved areas: the Caregiver Advise, Record, Enable (CARE) Act. J Aging Soc Policy. 2022;0(0):1-8. doi:10.1080/08959420.2022.2029272 35156557 PMC9374844

[27] Leykum LK, Penney LS, Dang S, . Recommendations to improve health outcomes through recognizing and supporting caregivers. J Gen Intern Med. 2022;37(5):1265-1269. doi:10.1007/s11606-021-07247-w 34981348 PMC8722428

[28] Pinquart M, Sörensen S. Ethnic differences in stressors, resources, and psychological outcomes of family caregiving: a meta-analysis. Gerontologist. 2005;45(1):90-106. doi:10.1093/geront/45.1.90 15695420

[29] Friedman EM, Tong PK, Rudin RS. The coronavirus pandemic highlights why family caregivers need to be integrated into the health care team and shows us how to make it happen. RAND Corporation. 2021. Accessed July 8, 2022. https://www.rand.org/pubs/perspectives/PEA1079-1.html

[30] National Academy for State Health Policy. RAISE Act state policy roadmap for family caregivers: family caregiver services and supports. May 20, 2022. Accessed July 9, 2022. https://www.nashp.org/family-caregiver-services-and-supports/

[31] National Academy for State Health Policy. RAISE Act state policy roadmap for family caregivers: public awareness and outreach to family caregivers. September 28, 2021. Accessed March 24, 2023. https://nashp.org/public-awareness-and-outreach-to-family-caregivers/

[32] Kutner G. AARP caregiver identification study. February 2001. Accessed March 24, 2023. https://assets.aarp.org/rgcenter/post-import/caregiver.pdf

[33] Salzberg CA, Kahn CN III, Foster NE, . Modernizing the HCAHPS Survey: recommendations from patient experience leaders. American Hospital Association. July 25, 2019. Accessed March 24, 2023. https://www.aha.org/guidesreports/2019-07-24-modernizing-hcahps-survey

