# Supplementary material for: Patient Experience at US Hospitals Following the Caregiver Advise, Record, Enable (CARE) Act
Source: JAMA Netw Open. 2023 May 1;6(5):e2311253. doi: 10.1001/jamanetworkopen.2023.11253 (PMC10152302; doi:10.1001/jamanetworkopen.2023.11253)
Supplement: Supplement 1. — eFigure 1. Flow Diagram of Sample Creation eTable 1. State-Level Passage of the CARE Act and Year Assigned in Analyses eTable 2. Timeline of State-Level Adoption of the CARE Act by State eTable 3. Hospital Consumer Assessment of Healthcare Providers and Systems (HCAHPS) Measurement Dates and Year Assignments for Quality Measures eTable 4. Overview of CARE Act Policy by State eTable 5. State-Level Caregiving Initiatives by State eFigure 2. Hospital Consumer Assessment of Healthcare Providers and Systems (HCAHPS) Survey Instrument eFigure 3. Frequency of Missingness for Hospitals Over the Study Period (2013-2019) eTable 6. Hospital Characteristics by CARE Act Passage Across All Years (2013-2019) eAppendix. Estimation Method Used in Callaway and Sant’Anna Differences-in-Differences Approach eTable 7. Regression Estimates for Parallel Pre-trends eFigure 5. Performance on HCAHPS, Unadjusted Means Over Time—Hospitals in CARE Act States vs. Non-CARE Act States eFigure 6. Estimates of Adjusted Differences in Care Transition Information Between Hospitals in CARE Act States vs Non-CARE Act States eTable 8. Differential Changes in Patient Experience and Readmissions Scores Between Hospitals in CARE Act and Non-CARE Act States by Time From Exposure eTable 9. Regression Estimates After Adding Interaction Term Between State and Year eTable 10. Regression Estimates Using Composite Scores of Top Box and Middle Box Responses [file jamanetwopen-e2311253-s001.pdf]

Supplemental Online Content

Lee CR, Taggert E, Coe NB, Chatterjee P. Patient experience at US hospitals following the Caregiver Advise, Record, Enable (CARE) Act. *JAMA Netw Open*. 2023;6(5):e2311253. doi:10.1001/jamanetworkopen.2023.11253

eFigure 1. Flow Diagram of Sample Creation
eTable 1. State-Level Passage of the CARE Act and Year Assigned in Analyses
eTable 2. Timeline of State-Level Adoption of the CARE Act by State
eTable 3. Hospital Consumer Assessment of Healthcare Providers and Systems (HCAHPS) Measurement Dates and Year Assignments for Quality Measures
eTable 4. Overview of CARE Act Policy by State
eTable 5. State-Level Caregiving Initiatives by State
eFigure 2. Hospital Consumer Assessment of Healthcare Providers and Systems (HCAHPS) Survey Instrument
eFigure 3. Frequency of Missingness for Hospitals Over the Study Period (2013-2019)
eTable 6. Hospital Characteristics by CARE Act Passage Across All Years (2013-2019)
eAppendix. Estimation Method Used in Callaway and Sant'Anna Differences-in-Differences Approach
eTable 7. Regression Estimates for Parallel Pre-trends
eFigure 5. Performance on HCAHPS, Unadjusted Means Over Time—Hospitals in CARE Act States vs. Non-CARE Act States
eFigure 6. Estimates of Adjusted Differences in Care Transition Information Between Hospitals in CARE Act States vs Non-CARE Act States
eTable 8. Differential Changes in Patient Experience and Readmissions Scores Between Hospitals in CARE Act and Non-CARE Act States by Time From Exposure
eTable 9. Regression Estimates After Adding Interaction Term Between State and Year
eTable 10. Regression Estimates Using Composite Scores of Top Box and Middle Box Responses

This supplemental material has been provided by the authors to give readers additional information about their work.

© 2023 Lee CR et al. *JAMA Network Open.*

eFigure 1. Flow Diagram of Sample Creation

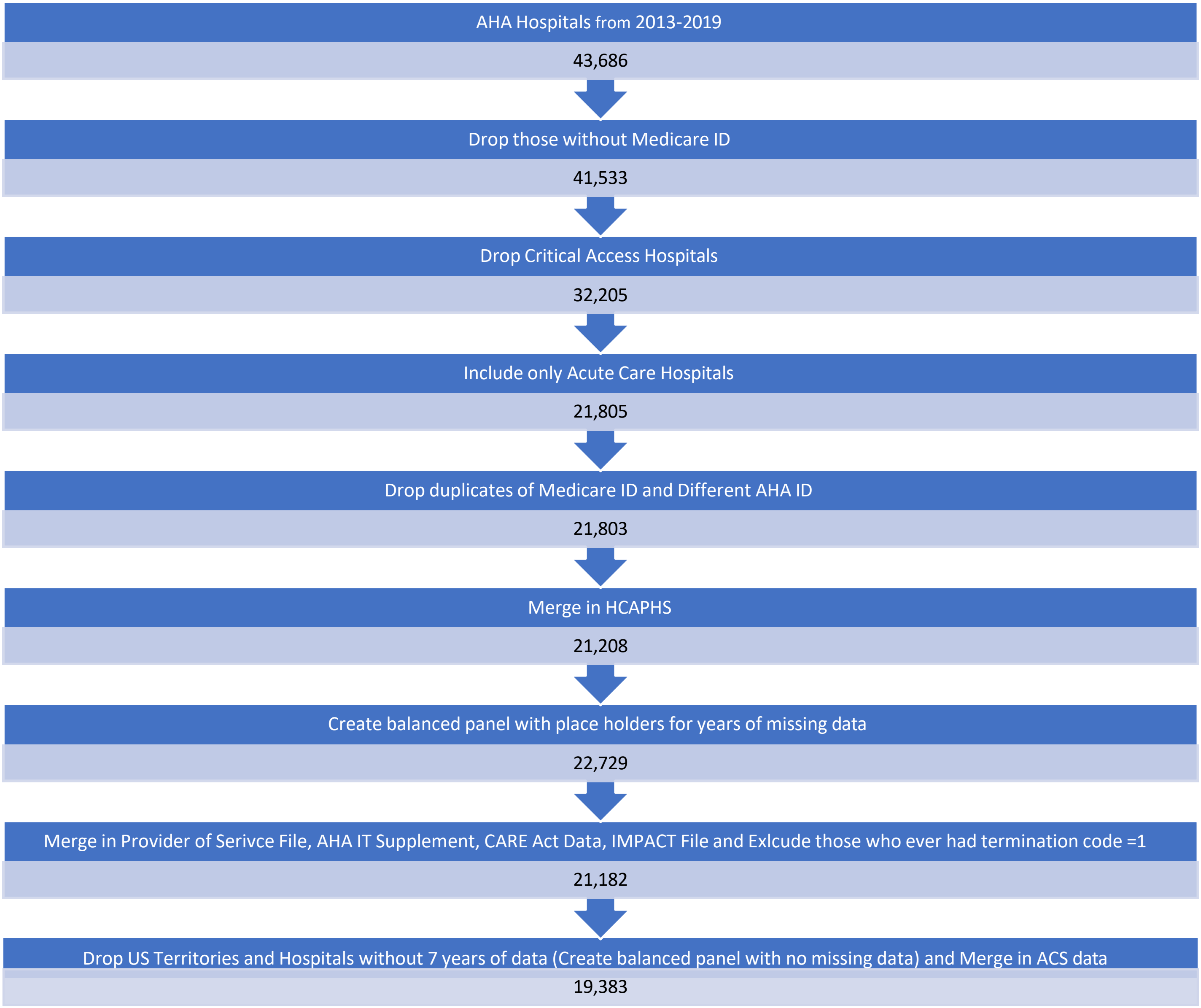

© 2023 Lee CR et al. *JAMA Network Open.*

eTable 1. State-Level Passage of the CARE Act and Year Assigned in Analyses

State	Enactment Date	Year Assignment in Analyses
AK	1/1/17	2018
AL	N/A	--
AR	7/22/15	2016
AZ	1/1/21	--
CA	1/1/16	2017
CO	5/8/15	2016
CT	10/1/15	2016
DC	7/1/2016	2017
DE	1/1/17	2018
FL	N/A	--
GA	7/1/22	--
HI	7/1/17	2018
IA	7/1/19	--
ID	N/A	--
IL	1/27/16	2017
IN	1/1/16	2017
KS	7/1/18	2019
KY	6/29/17	2018
LA	8/1/16	2017
MA	11/8/17	2018
MD	10/1/16	2017
ME	10/15/15	2016
MI	7/12/16	2017
MN	1/1/17	2018
MO	8/28/18	2019
MS	7/1/15	2016
MT	10/1/17	2018
NC	N/A	--
ND	8/1/19	--
NE	3/30/16	2017
NH	1/1/16	2017

© 2023 Lee CR et al. *JAMA Network Open.*

NJ	5/12/15	2016
NM	6/17/15	2016
NV	10/1/15	2016
NY	4/23/16	2017
OH	3/21/17	2018
OK	11/5/14	2015
OR	1/1/16	2017
PA	4/20/17	2018
RI	3/14/17	2018
SC	N/A	--
SD	N/A	--
TN	6/6/19	--
TX	5/26/17	2018
UT	2/10/16	2017
VA	7/1/15	2016
VT	N/A	--
WA	6/9/16	2017
WI	N/A	--
WV	6/8/15	2016
WY	7/1/16	2017

For all states that passed the CARE Act, the dates reflect when the policy was passed (Last updated by AARP here in 10/2020). The corresponding year assignment in analyses lags by one year to account for potential delay in policy implementation within hospitals.

© 2023 Lee CR et al. *JAMA Network Open.*

eTable 2. Timeline of State-Level Adoption of the CARE Act by State

			WY (Jul)						
			DC (Jul)						
			WA (Jun)						
			VI (Mar)						
			UT (Feb)	TX (May)					
		WV (Jun)	OR (Jan)	RI (Mar)					
		VA (Jul)	NY (Apr)	PA (Apr)					
		NM (Jun)	NH (Jan)	OH (Mar)					
		NJ (May)	NE (Mar)	MT (Oct)					
		NV (Oct)	MI (Jul)	MN (Jan)					
		MS (Jul)	MD (Oct)	MA (Nov)					
		ME (Oct)	LA (Aug)	KY (Jun)					
		CT (Oct)	IN (Jan)	HI (Jul)		TN (Jun)			
		CO (May)	IL (Jul)	DE (Jan)	MO (Aug)	ND (Aug)			
	OK (Nov)	AR (Jul)	CA (Jan)	AK (Jan)	KS (Jul)	IA (Jul)		AZ (Jan)	GA (Jul)
**2013**	**2014**	**2015**	**2016**	**2017**	**2018**	**2019**	**2020**	**2021**	**2022**

Never-passed States: AL, FL, ID, NC, SC, SD, VT, WI

eTable 3. Hospital Consumer Assessment of Healthcare Providers and Systems (HCAHPS) Measurement Dates and Year Assignments for Quality Measures

Quality Measure	Hospital Compare Reporting Year	Measurement Dates	Year Assigned for Analysis
HCAHPS	2014	1/1/2013-12/31/2013	2013
	2015	1/1/2014-12/31/2014	2014
	2016	1/1/2015-12/31/2015	2015
	2017	1/1/2016-12/31/2016	2016
	2018	1/1/2017-12/31/2017	2017
	2019	1/1/2018-12/31/2018	2018
	2020	1/1/2019-12/31/2019	2019

For each reporting year of HCAHPS, measurements represent the prior year and were assigned as such in the analyses.

© 2023 Lee CR et al. *JAMA Network Open.*

eTable 4. Overview of CARE Act Policy by State

For all states that passed the CARE Act, no hospital was held liable for identifying a caregiver prior to executing patient discharge. No state required hospitals to be monitored at the state level except for Pennsylvania, which requires the Legislative Budget and Finance Committee to conduct a study no later than three years after the effective date of Section 9 of Pennsylvania's CARE Act legislation. This study is regarding the impact of the CARE act in certain patient outcomes, including, but not limited to, hospital readmissions.

		CARE Act Legislation						
State		Date of Legalization	First year of analysis coded as enacted	Elements of advise, record, & enable present[a-c]	Alternatives given for incapacitated patients	Hospital Liable for Caregiver Training	Responsibility of hospital to find caregiver prior to discharge	State Monitoring
Alaska	AK	1/1/17	2018	X	X	No	No	No
Arizona[d]	AZ	1/1/21	N/A	N/A	N/A	N/A	N/A	N/A
Arkansas	AR	7/22/15	2016	X		No	No	No
California	CA	1/1/16	2017	X	X	No	No	No
Colorado	CO	5/8/15	2016	X	X	No	No	No
Connecticut	CT	10/1/15	2016	X		No	No	No
Delaware	DE	1/1/17	2018	X		No	No	No
Georgia	GA	7/1/22	N/A	X		No	No	No
Hawaii	HI	7/1/17	2018	X		No	No	No
Illinois	IL	1/27/16	2017	X	X	No	No	No
Indiana	IN	1/1/16	2017	X		No	No	No
Iowa	IA	7/1/19	2020	X	X	No	No	No
Kansas	KS	7/1/18	2019	X		No	No	No
Kentucky	KY	6/29/17	2018	X	X	No	No	No
Louisiana	LA	8/1/16	2017	X	X	No	No	No
Maine	ME	10/15/15	2016	X	X	No	No	No
Maryland	MD	10/1/16	2017	X		No	No	No
Massachusetts	MA	11/8/17	2018	X	X	No	No	No
Michigan	MI	7/12/16	2016	X	X	No	No	No
Minnesota	MN	1/1/17	2018	X	X	No	No	No
Mississippi	MS	7/1/15	2016	X	X	No	No	No
Missouri	MO	8/28/18	2019	X		No	No	No
Montana	MT	10/1/17	2018	X		No	No	No
Nebraska	NE	3/30/16	2017	X	X	No	No	No

© 2023 Lee CR et al. *JAMA Network Open.*

Nevada	NV	10/1/15	2016	X	X	No	No	No
New Hampshire	NH	1/1/16	2017	X	X	No	No	No
New Jersey	NJ	5/12/15	2016	X	X	No	No	No
New Mexico	NM	6/17/15	2016	X		No	No	No
New York	NY	4/23/16	2017	X	X	No	No	No
North Dakota	ND	8/1/19	2020	X	X	No	No	No
Ohio	OH	3/21/17	2018	X	X	No	No	No
Oklahoma	OK	11/5/14	2015	X	X	No	No	No
Oregon	OR	1/1/16	2017	X		No	No	No
Pennsylvania	PA	4/20/17	2018	X	X	No	No	Yes
Rhode Island	RI	3/14/17	2018	X	X	No	No	No
Tennessee[d]	TN	6/6/19	N/A	N/A	N/A	N/A	N/A	N/A
Texas	TX	5/26/17	2018	X		No	No	No
Utah	UT	2/10/16	2017	X		No	No	No
Virginia	VA	7/1/15	2016	X		No	No	No
Washington	WA	6/9/16	2017	X		No	No	No
West Virginia	WV	6/8/15	2016	X	X	No	No	No
Wyoming	WY	7/1/16	2017	X	X	No	No	No
Alabama	AL	--						
Florida	FL	--						
Idaho	ID	--						
North Carolina	NC	--						
South Carolina	SC	--						
South Dakota	SD	--						
Vermont	VT	--						
Wisconsin	WI	--						

[a]Advise is defined as notification to individuals of their opportunity to identify a family caregiver.
[b]Record is defined as documentation of the caregiver's name and contact information in the health record (with the patient's permission).
[c]Enable is defined as notification of discharge, providing training instructions for medical-nursing tasks, or providing written materials, such as discharge summary summarizing reason for and treatments provided during hospitalization.
[d]The researchers were unable to CARE Act legislation for these states.

© 2023 Lee CR et al. *JAMA Network Open.*

eTable 5. State-Level Caregiving Initiatives by State

State		State-level Caregiving Initiatives			
		Caregiver Support Programs	Workplace Benefits for Family Caregivers	Home & Community Based Services	Other
Alaska	AK	X	X	X	
Arizona	AZ	X	X	X	X
Arkansas	AR	X	X	X	
California	CA	X	X	X	X
Colorado	CO	X	X	X	X
Connecticut	CT	X	X	X	
Delaware	DE	X	X	X	
Georgia	GA	X		X	
Hawaii	HI	X	X	X	X
Illinois	IL	X		X	
Indiana	IN	X		X	
Iowa	IA	X	X		X
Kansas	KS	X	X	X	
Kentucky	KY	X		X	
Louisiana	LA	X		X	
Maine	ME	X	X	X	
Maryland	MD	X		X	X
Massachusetts	MA	X	X	X	
Michigan	MI	X	X	X	
Minnesota	MN	X	X	X	X
Mississippi	MS	X		X	
Missouri	MO	X		X	X
Montana	MT	X		X	
Nebraska	NE	X	X	X	X
Nevada	NV	X	X	X	
New Hampshire	NH	X	X	X	
New Jersey	NJ	X	X	X	
New Mexico	NM	X		X	
New York	NY	X	X	X	X
North Dakota	ND	X		X	
Ohio	OH	X		X	

© 2023 Lee CR et al. *JAMA Network Open.*

Oklahoma	OK	X	X	X	
Oregon	OR	X	X	X	
Pennsylvania	PA	X	X	X	X
Rhode Island	RI		X	X	
Tennessee	TN	X		X	
Texas	TX	X	X	X	X
Utah	UT	X		X	
Virginia	VA	X		X	
Washington	WA	X	X	X	
West Virginia	WV	X		X	
Wyoming	WY	X		X	X
Alabama	AL	X		X	
Florida	FL	X		X	
Idaho	ID	X	X	X	
North Carolina	NC	X		X	X
South Carolina	SC	X	X	X	
South Dakota	SD	X		X	
Vermont	VT	X		X	
Wisconsin	WI	X		X	

© 2023 Lee CR et al. *JAMA Network Open.*

eFigure 2. Hospital Consumer Assessment of Healthcare Providers and Systems (HCAHPS) Survey Instrument

HCAHPS Survey

SURVEY INSTRUCTIONS

- You should only fill out this survey if you were the patient during the hospital stay named in the cover letter. Do not fill out this survey if you were not the patient.
- Answer all the questions by checking the box to the left of your answer.
- You are sometimes told to skip over some questions in this survey. When this happens you will see an arrow with a note that tells you what question to answer next, like this:

  ☐ Yes
  ☑ No ➔ ***If No, Go to Question 1***

You may notice a number on the survey. This number is used to let us know if you returned your survey so we don't have to send you reminders.
Please note: Questions 1-29 in this survey are part of a national initiative to measure the quality of care in hospitals. OMB #0938-0981 (Expires September 30, 2024)

Please answer the questions in this survey about your stay at the hospital named on the cover letter. Do not include any other hospital stays in your answers.

YOUR CARE FROM NURSES

1. During this hospital stay, how often did nurses treat you with courtesy and respect?

1☐ Never
2☐ Sometimes
3☐ Usually
4☐ Always

2. During this hospital stay, how often did nurses listen carefully to you?

1☐ Never
2☐ Sometimes
3☐ Usually
4☐ Always

3. During this hospital stay, how often did nurses explain things in a way you could understand?

1☐ Never
2☐ Sometimes
3☐ Usually
4☐ Always

4. During this hospital stay, after you pressed the call button, how often did you get help as soon as you wanted it?

1☐ Never
2☐ Sometimes
3☐ Usually
4☐ Always
9☐ I never pressed the call button

© 2023 Lee CR et al. *JAMA Network Open.*

YOUR CARE FROM DOCTORS

5. **During this hospital stay, how often did doctors treat you with <u>courtesy and respect</u>?**

   1☐ Never
   2☐ Sometimes
   3☐ Usually
   4☐ Always

6. **During this hospital stay, how often did doctors <u>listen carefully to you</u>?**

   1☐ Never
   2☐ Sometimes
   3☐ Usually
   4☐ Always

7. **During this hospital stay, how often did doctors <u>explain things</u> in a way you could understand?**

   1☐ Never
   2☐ Sometimes
   3☐ Usually
   4☐ Always

THE HOSPITAL ENVIRONMENT

8. **During this hospital stay, how often were your room and bathroom kept clean?**

   1☐ Never
   2☐ Sometimes
   3☐ Usually
   4☐ Always

9. **During this hospital stay, how often was the area around your room quiet at night?**

   1☐ Never
   2☐ Sometimes
   3☐ Usually
   4☐ Always

YOUR EXPERIENCES IN THIS HOSPITAL

10. **During this hospital stay, did you need help from nurses or other hospital staff in getting to the bathroom or in using a bedpan?**

   1☐ Yes
   2☐ No ➔ **If No, Go to Question 12**

11. **How often did you get help in getting to the bathroom or in using a bedpan as soon as you wanted?**

   1☐ Never
   2☐ Sometimes
   3☐ Usually
   4☐ Always

12. **During this hospital stay, were you given any medicine that you had not taken before?**

   1☐ Yes
   2☐ No ➔ **If No, Go to Question 15**

13. **Before giving you any new medicine, how often did hospital staff tell you what the medicine was for?**

   1☐ Never
   2☐ Sometimes
   3☐ Usually
   4☐ Always

14. **Before giving you any new medicine, how often did hospital staff describe possible side effects in a way you could understand?**

   1☐ Never
   2☐ Sometimes
   3☐ Usually
   4☐ Always

© 2023 Lee CR et al. *JAMA Network Open.*

WHEN YOU LEFT THE HOSPITAL

15. After you left the hospital, did you go directly to your own home, to someone else's home, or to another health facility?

1☐ Own home
2☐ Someone else's home
3☐ Another health facility ➔ **If Another, Go to Question 18**

16. During this hospital stay, did doctors, nurses or other hospital staff talk with you about whether you would have the help you needed when you left the hospital?

1☐ Yes
2☐ No

17. During this hospital stay, did you get information in writing about what symptoms or health problems to look out for after you left the hospital?

1☐ Yes
2☐ No

OVERALL RATING OF HOSPITAL

Please answer the following questions about your stay at the hospital named on the cover letter. Do not include any other hospital stays in your answers.

18. Using any number from 0 to 10, where 0 is the worst hospital possible and 10 is the best hospital possible, what number would you use to rate this hospital during your stay?

0☐ 0 Worst hospital possible
1☐ 1
2☐ 2
3☐ 3
4☐ 4
5☐ 5
6☐ 6
7☐ 7
8☐ 8
9☐ 9
10☐ 10 Best hospital possible

19. Would you recommend this hospital to your friends and family?

1☐ Definitely no
2☐ Probably no
3☐ Probably yes
4☐ Definitely yes

UNDERSTANDING YOUR CARE WHEN YOU LEFT THE HOSPITAL

20. During this hospital stay, staff took my preferences and those of my family or caregiver into account in deciding what my health care needs would be when I left.

1☐ Strongly disagree
2☐ Disagree
3☐ Agree
4☐ Strongly agree

© 2023 Lee CR et al. *JAMA Network Open.*

21. **When I left the hospital, I had a good understanding of the things I was responsible for in managing my health.**

- [1] ☐ Strongly disagree
- [2] ☐ Disagree
- [3] ☐ Agree
- [4] ☐ Strongly agree

22. **When I left the hospital, I clearly understood the purpose for taking each of my medications.**

- [1] ☐ Strongly disagree
- [2] ☐ Disagree
- [3] ☐ Agree
- [4] ☐ Strongly agree
- [5] ☐ I was not given any medication when I left the hospital

ABOUT YOU

There are only a few remaining items left.

23. **During this hospital stay, were you admitted to this hospital through the Emergency Room?**

- [1] ☐ Yes
- [2] ☐ No

24. **In general, how would you rate your overall health?**

- [1] ☐ Excellent
- [2] ☐ Very good
- [3] ☐ Good
- [4] ☐ Fair
- [5] ☐ Poor

25. **In general, how would you rate your overall <u>mental or emotional health</u>?**

- [1] ☐ Excellent
- [2] ☐ Very good
- [3] ☐ Good
- [4] ☐ Fair
- [5] ☐ Poor

26. **What is the highest grade or level of school that you have <u>completed</u>?**

- [1] ☐ 8th grade or less
- [2] ☐ Some high school, but did not graduate
- [3] ☐ High school graduate or GED
- [4] ☐ Some college or 2-year degree
- [5] ☐ 4-year college graduate
- [6] ☐ More than 4-year college degree

27. **Are you of Spanish, Hispanic or Latino origin or descent?**

- [1] ☐ No, not Spanish/Hispanic/Latino
- [2] ☐ Yes, Puerto Rican
- [3] ☐ Yes, Mexican, Mexican American, Chicano
- [4] ☐ Yes, Cuban
- [5] ☐ Yes, other Spanish/Hispanic/Latino

28. **What is your race? Please choose one or more.**

- [1] ☐ White
- [2] ☐ Black or African American
- [3] ☐ Asian
- [4] ☐ Native Hawaiian or other Pacific Islander
- [5] ☐ American Indian or Alaska Native

© 2023 Lee CR et al. *JAMA Network Open.*

29. What language do you mainly speak at home?

- 1☐ English
- 2☐ Spanish
- 3☐ Chinese
- 4☐ Russian
- 5☐ Vietnamese
- 6☐ Portuguese
- 7☐ German
- 8☐ Tagalog
- 9☐ Arabic
- 20☐ Some other language (please print): ______________________

NOTE: IF HOSPITAL-SPECIFIC SUPPLEMENTAL QUESTION(S) ARE ADDED, THE MANDATORY TRANSITION STATEMENT MUST BE PLACED IMMEDIATELY BEFORE THE SUPPLEMENTAL QUESTION(S).

THANK YOU

Please return the completed survey in the postage-paid envelope.

[NAME OF SURVEY VENDOR OR SELF-ADMINISTERING HOSPITAL]

[RETURN ADDRESS OF SURVEY VENDOR OR SELF-ADMINISTERING HOSPITAL]

Questions 1-19 and 23-29 are part of the HCAHPS Survey and are works of the U.S. Government. These HCAHPS questions are in the public domain and therefore are NOT subject to U.S. copyright laws. The three Care Transitions Measure® questions (Questions 20-22) are copyright of Eric A. Coleman, MD, MPH, all rights reserved.

© 2023 Lee CR et al. *JAMA Network Open.*

eFigure 3. Frequency of Missingness for Hospitals Over the Study Period (2013-2019)
There were 256 hospitals (out of 3019 total hospitals, 8.48%) that demonstrated missingness over the study period (2013-2019). Years of missing data are the result of unobserved years of reporting into HCAPHS or responses not being publicly available in the data. See table below.

Years of Complete Data	# Hospitals	% Hospitals	Details on Timing of Missingness
0	18*	0.60	
1	54	1.79	
2	26	0.86	
3	25	0.83	
4	23	0.76	
5	31	1.03	
6	79	2.62	
7	2763	91.52	Complete panel of observations
Total	3019	100%	

# Missing Outcomes (Range 0-7)	# Hospital-Years	% Hospital-Years
0	20,243	95.79
1	2	0.01
3	1	0.00
7	887	4.20

Survey responses were consistent within-year reporting. For example, if a hospital had at least 1 survey response, they were highly likely to have all responses in that year. We examined 7 outcome variables at the hospital-year level. Of 21,133 hospital-year observations, 20,243 (95.79%) had complete data across the 7 outcomes, 887 (4.20%) were missing all 7 responses in a given year, 2 (0.01%) hospitals were missing 1 outcomes in 2014 and 1 (<0.001%) hospital in 2015 was missing 3 outcomes. See tables above.

Year	# (%) Hospitals Missing 7 Outcomes	# (%) Hospitals Missing 3 Outcomes	# (%) Hospitals Missing 1 Outcome
2013	122 (0.58)	0	0
2014	139 (0.66)	0	2
2015	125 (0.59)	1	0
2016	127 (0.60)	0	0
2017	121 (0.57)	0	0
2018	108 (0.51)	0	0
2019	145 (0.69)	0	0
Total	887 (4.20)	1	2

© 2023 Lee CR et al. *JAMA Network Open.*

Year	Total N=21,113<br># (%) Hospitals Missing Any Outcomes	Non-CARE Act N=5516<br># (%) Hospitals Missing Any Outcomes	CARE Act N=15617<br># (%) Hospitals Missing Any Outcomes
2013	122 (4.04)	35 (4.44)	87 (3.9)
2014	141 (4.67)	63 (7.99)	78 (3.5)
2015	126 (4.17)	63 (7.99)	63 (2.82)
2016	127 (4.21)	63 (7.99)	64 (2.87)
2017	121 (4.01)	64 (8.12)	57 (2.55)
2018	108 (3.58)	62 (7.87)	46 (2.06)
2019	145 (4.80)	72 (9.14)	73 (3.27)
Total hospital-years	890 (4.20)	422 (7.7)	468 (3.0)

We examined hospital characteristics by missingness across all years in the study period. Given hospitals characteristics such as hospital ownership or size may be potential explanations for why hospitals may not have had data available or have had data missing during the study period. Several observations that were missing data for the outcomes were also missing from the American Hospital Association (AHA) dataset from which we received information about hospital characteristics.

	Hospital-year observations, No. (%)	
**Characteristics**	**Missing data (n= 890)**	**No missing data (n=20,243)**
Urban		
Rural	132 (18.0%)	5489 (27.3%)
Urban	600 (82.0%)	14,651 (72.7%)
Size		
Small (<100)	736 (82.7%)	5878 (29.0%)
Medium (100-399)	143 (16.1%)	11,269 (55.7%)
Large (>400)	11 (1.2%)	3096 (15.3%)
Profit		
Government	136 (15.3%)	2856 (14.1%)
Nonprofit	321 (36.1%)	13,314 (65.8%)
For-profit	429 (48.2%)	4073 (20.1%)
Other	4 (0.4%)	0 (0%)
Region		
Northeast	52 (7.4%)	3149 (15.6%)
Midwest	139 (19.7%)	4732 (23.4%)
South	397 (56.4%)	8420 (41.6%)
West	116 (16.5%)	3940 (19.5%)
Teaching		
Non-teaching	779 (87.5%)	12,458 (61.5%)
Case Mix Index, mean (SD)	1.7 (0.7)	1.5 (0.3)
Proportion Medicare days, mean (SD)	51.1 (18.4)	51.4 (13.4)
Proportion Medicaid days, mean (SD)	12.8 (14.4)	20.6 (12.6)

© 2023 Lee CR et al. *JAMA Network Open.*

eTable 6. Hospital Characteristics by CARE Act Status Across All Years (2013-2019)

Characteristics	Hospital-year observations, No. (%) Non-CARE Act (n=4025)	CARE Act (n=15358)
Hospitals, No.	575	2188
Size		
Small (<100)	1110 (27.6%)	4171 (27.2%)
Medium (100-399)	2213 (55.0%)	8821 (57.4%)
Large (≥400)	702 (17.4%)	2366 (15.4%)
Profit Status		
Government	753 (18.7%)	1968 (12.8%)
Nonprofit	2194 (54.5%)	10717 (69.8%)
For-profit	1078 (26.8%)	2673 (17.4%)
Region		
Northeast	42 (1.0%)	3031 (19.8%)
Midwest	490 (12.2%)	4081 (26.6%)
South	3108 (77.2%)	4872 (31.5%)
West	385 (9.6%)	3374 (22.0%)
Urbanicity		
Rural	1109 (27.6%)	4141 (27.1%)
Urban	2914 (72.4%)	11156 (72.9%)
Teaching Status		
Non-teaching	262 (6.5%)	1273 (8.5%)
Teaching	3763 (93.5%)	14043 (91.7%)
Electronic health record	2553 (99.3%)	9827 (98.5%)
Case Mix Index, mean (SD)	1.5 (0.3)	1.5 (0.3)
% Medicare days, mean (SD)[a]	51.9 (13.1)	51.4 (13.3)
% Medicaid days, mean (SD)[b]	19.6 (10.8)	21.1 (13.0)
County-level fraction over 65, mean (SD)	0.2 (0.1)	0.1 (0.0)

[a]Medicare inpatient days/total inpatient days x 100.
[b]Medicaid inpatient days/total inpatient days x 100.
Abbreviation: CARE, Caregiver Advise Record Enable.

© 2023 Lee CR et al. *JAMA Network Open.*

eAppendix. Estimation Method Used in Callaway and Sant'Anna Differences-in-Differences Approach

In the Callaway and Sant'Anna (CS) differences-in-differences (DiD) approach, we chose the outcome regression estimation method to identify our estimators. While each of the potential CS DiD estimation methods are equivalent in terms of identification, the outcome regression method allowed for more easily interpretable inferences after conditioning our model on relevant covariates. We also analyzed our data using the doubly-robust estimators, which includes both inverse probability weighting and outcomes regression. We found that our main findings were consistent using this estimation method, therefore, we did not include in this paper.

© 2023 Lee CR et al. *JAMA Network Open.*

eTable 7. Regression Estimates for Parallel Pre-trends

Quality Measure	Pre-2016 Unadjusted Mean (SD)	Coefficient for Pre-trend	P
Nurse communication	78.0 (0.03)	-0.03	0.76
Physician communication	80.3 (0.03)	-0.09	0.15
Communication about Medications	62.9 (0.01)	-0.20	0.25
Discharge information	86.0 (0.06)	-0.03	0.73
Care Transition Information	50.5 (0.10)	-0.15	0.32
Overall Hospital Rating	69.5 (0.15)	-0.03	0.78
Recommend Hospital to Others	70.1 (0.03)	-0.05	0.66

© 2023 Lee CR et al. *JAMA Network Open.*

eFigure 5. Performance on HCAHPS Unadjusted Means Over Time—Hospitals in CARE Act States vs. Non-CARE Act States

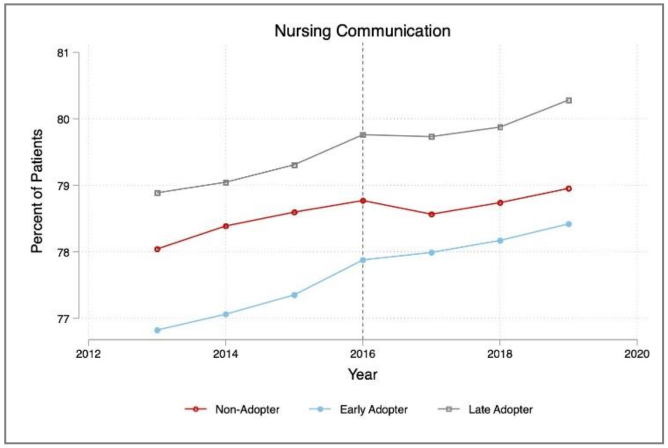

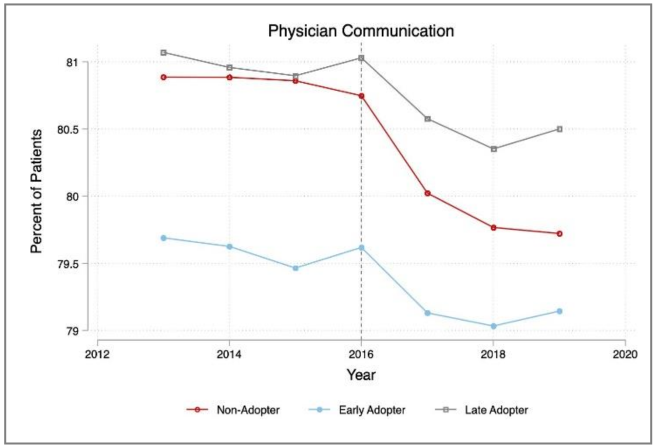

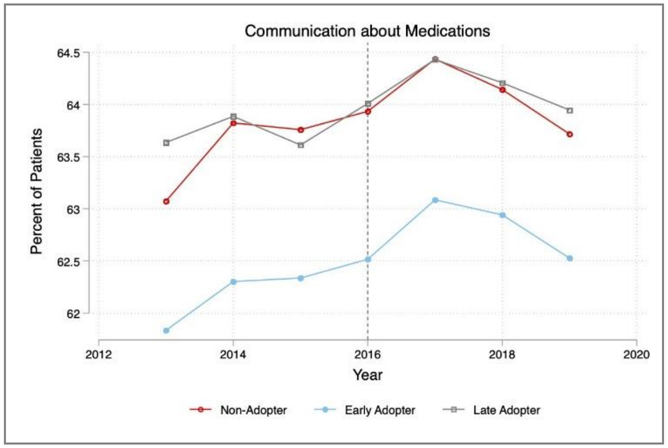

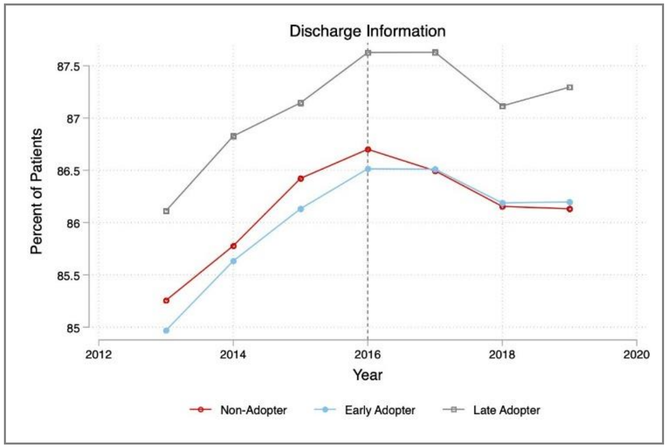

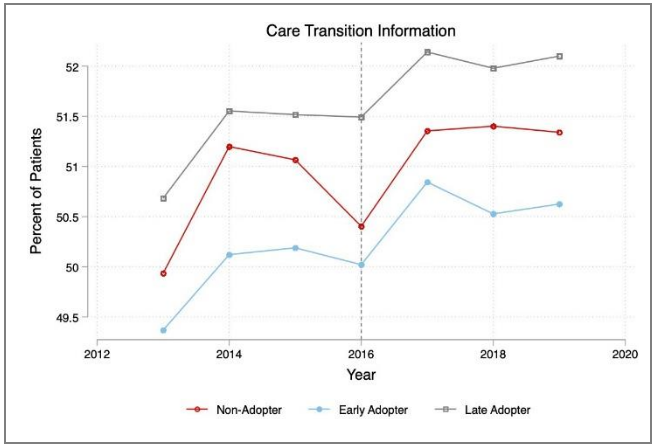

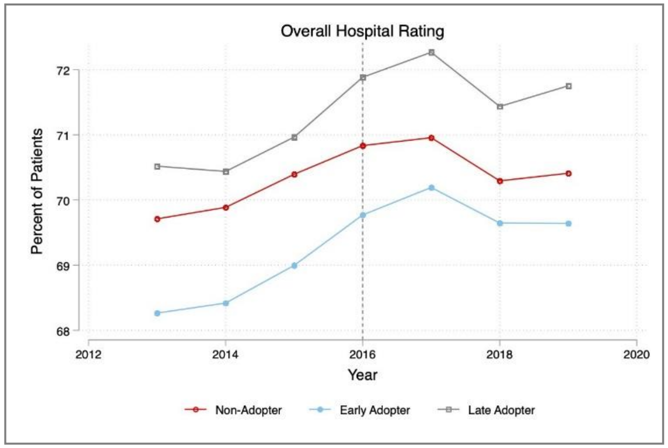

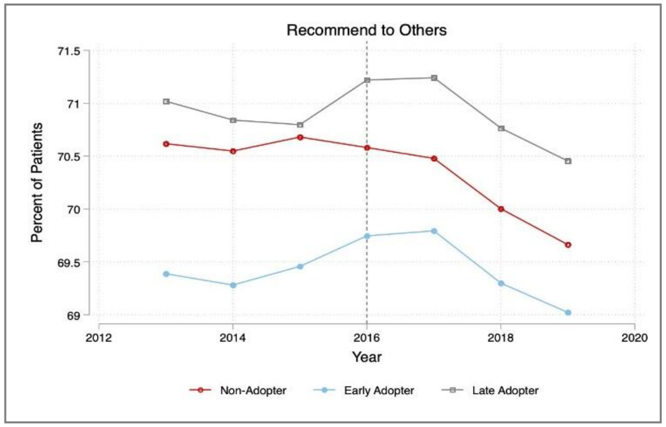

© 2023 Lee CR et al. *JAMA Network Open.*

eFigure 6. **Estimates of Adjusted Differences in Care Transition Information Between Hospitals in CARE Act States vs Non-CARE Act States**

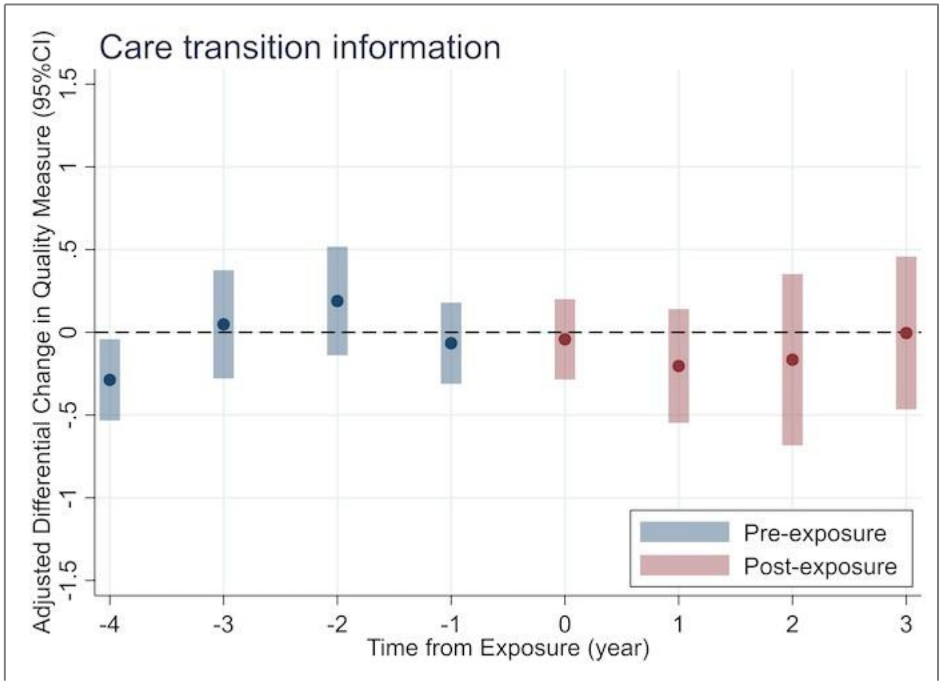

The absolute adjusted difference in quality measure between hospitals located in exposed states and not yet exposed states for each outcome are plotted. The x-axis represents the number of years relative to CARE Act passage with event years greater than 4 years before exposure and 3 years after exposure excluded from the analyses. The sample consisted of 19,383 hospital-year observations and represented 38 exposed and 13 unexposed states. All models adjusted for hospital characteristics (number of beds, ownership type), county-level proportion of 65 and older, and year- and state-fixed effects to reflect the level of the policy exposure.

© 2023 Lee CR et al. *JAMA Network Open.*

eTable 8. Differential Changes in Patient Experience Between Hospitals in CARE Act and Non-CARE Act States by Time From Exposure

Time from Exposure (year)	Nursing Communication (95% CI)[a]	Physician Communication (95% CI)[a]	Communication about Medications (95% CI)[a]	Discharge Information (95% CI)[a]	Care Transition Information (95% CI)[a]	Overall Hospital Rating (95% CI)[a]	Recommend Hospital to Others (95% CI)[a]
**-4**	0.02 (-0.25, 0.29)	-0.09 (-0.36, 0.19)	-0.29 (-0.64, 0.06)	0.13 (-0.13, 0.40)	-0.29* (-0.53, -0.04)	-0.09 (-0.52, 0.33)	-0.19 (-0.54, 0.16)
**-3**	0.13 (-0.06, 0.32)	0.16 (-0.03, 0.37)	0.10 (-0.24, 0.45)	<0.01 (-0.17, 0.16)	0.05 (-0.28, 0.38)	0.09 (-0.16, 0.35)	0.08 (-0.14, 0.30)
**-2**	0.04 (-0.15, 0.22)	-0.10 (-0.27, 0.06)	0.17 (-0.09, 0.43)	0.08 (-0.07, 0.24)	0.19 (-0.14, 0.52)	0.15 (-0.12, 0.42)	0.14 (-0.12, 0.40)
**-1**	0.19* (0.04, 0.33)	0.11 (-0.08, 0.29)	-0.07 (-0.33, 0.21)	0.02 (-0.11, 0.14)	-0.07 (-0.31, 0.18)	0.11 (-0.13, 0.34)	0.10 (-0.12, 0.33)
**0**	0.11 (-0.06, 0.28)	0.12 (0.02, 0.26)	-0.05 (-0.25, 0.15)	0.10 (-0.06, 0.26)	-0.04 (-0.29, 0.20)	0.07 (-0.26, 0.41)	-0.002 (-0.33, 0.32)
**1**	0.31** (0.08, 0.53)	0.40* (0.20, 0.59)	0.05 (-0.27, 0.38)	0.23 (-0.02, 0.48)	-0.20 (-0.55, 0.14)	0.46* (0.11, 0.80)	0.21 (-0.16, 0.57)
**2**	0.35* (0.01, 0.68)	0.47* (0.11, 0.84)	0.20 (-0.41, 0.80)	0.30 (-0.03, 0.63)	-0.17 (-0.68, 0.35)	0.57 (-0.04, 1.18)	0.44 (-0.24, 1.12)
**3**	0.54* (0.10, 0.98)	0.59** (0.17, 1.0)	-0.44 (-1.5, 0.60)	0.07 (-0.33, 0.47)	-0.004 (-0.47, 0.46)	0.72 (-0.11, 1.56	0.82* (0.10, 1.55)

$^{*}p \leq 0.05$ $^{**}p \leq 0.01$ $^{***}p \leq 0.001$

[a]Differential change represents the estimated percentage point difference between hospitals in CARE Act states compared to those in Non-CARE Act states after adjusting for number of beds, ownership type and proportion of 65 years and older characteristics. We also adjusted for hospital- and year-fixed effects, and clustered standard errors at the state to reflect the level of the policy exposure

© 2023 Lee CR et al. *JAMA Network Open.*

Sensitivity Analyses

eTable 9. Regression Estimates After Adding Interaction Term Between State and Year

Quality measure	Unadjusted mean (SD), %	Adjusted differential change[a] (95% CI)	P
**Patient communication**			
Nursing communication	78.4 (0.42)	0.18 (0.7, 0.28)	0.001
Doctor communication	80.0 (0.19)	0.46 (0.34, 0.57)	0.00
Communication on medications	63.2 (0.20)	-0.43 (-0.59, -0.27)	0.00
Discharge information	86.4 (0.22)	-0.03 (-0.11, 0.05)	0.41
Care transition information	50.8 (0.30)	-0.16 (-0.33,0.01)	0.07
**Patient experience**			
Overall hospital rating	70.1 (0.41)	0.24 (0.07, 0.41)	0.006
Recommend hospital to others	70.1 (0.06)	0.15 (-0.00, 0.30)	0.06

[a]Differential change represents the estimated percentage point difference between hospitals in CARE Act states compared to those in Non-CARE Act states after adjusting for number of beds, ownership type and proportion of 65 years and older characteristics. We also adjusted for hospital- and year-fixed effects, and clustered standard errors at the state to reflect the level of the policy exposure.

© 2023 Lee CR et al. *JAMA Network Open.*

eTable 10. Regression Estimates Using Composite Scores of Top Box and Middle Box Responses

Quality measure	Unadjusted mean (SD), %	Adjusted differential change[a] (95% CI)	P
**Patient communication**			
Nursing communication[b]	95.2 (0.02)	0.10 (0.05, 0.14)	0.00
Doctor communication[c]	95.2 (0.20)	0.10 (0.02,0.17)	0.01
Communication on medications[d]	81.1 (0.21)	0.03 (-0.12,0.19)	0.68
Discharge information	NA	NA	NA
Care transition information[e]	94.3 (0.08)	-0.008 (-0.8,0.06)	0.82
**Patient experience**			
Overall hospital rating[f]	91.6 (0.05)	0.15 (0.06,0.23)	<0.01
Recommend hospital to others[g]	94.5 (0.09)	0.06 (-0.01,0.14)	0.10

[a]Differential change represents the estimated percentage point difference between hospitals in CARE Act states compared to those in Non-CARE Act states after adjusting for number of beds, ownership type and proportion of 65 years and older characteristics. We also adjusted for hospital- and year-fixed effects, and clustered standard errors at the state to reflect the level of the policy exposure
[b]Nurse communication – Percent of patients in a hospital who reported that they \`Always' or \`Usually' communicated well with nurses
[c]Doctor communication – Percent of patients in a hospital who reported that they \`Always' or \`Usually' communicated well with physicians
[d]Communication about medications -- Percent of patients in a hospital who reported that they \`Always' or \`Usually' communicated about medications
[e]Care transition information – Percent of patients in a hospital who reported that they \`Strongly agree' or \`Agree' they received care transition information
[f]Overall hospital rating – Percent of patients in a hospital who assigned a score of 7 or 8, or a score of 9 or 10 on 10-point scale
[g]Recommend hospital to others – Percent of patients in a hospital who responded "definitely yes" or "probably yes" to whether they would recommend the hospital others

© 2023 Lee CR et al. *JAMA Network Open.*
