# Supplementary material for: Patient Experience at US Hospitals Following the Caregiver Advise, Record, Enable (CARE) Act
Source: JAMA Netw Open. 2023 May 1;6(5):e2311253. doi: 10.1001/jamanetworkopen.2023.11253 (PMC10152302; doi:10.1001/jamanetworkopen.2023.11253)
Supplement: Supplement 2. — Data Sharing Statement [file jamanetwopen-e2311253-s002.pdf]

Data Sharing Statement

Lee. Patient Experience at US Hospitals Following the Caregiver Advise, Record, Enable (CARE) Act. *JAMA Netw Open*. Published May 01, 2023.
doi:10.1001/jamanetworkopen.2023.11253

Data
Data available: Yes
Data types: Deidentified participant data
How to access data: Data will be available upon request via email to courtney.lee@pennmedicine.upenn.edu.
When available: With publication

Supporting Documents
Document types: Statistical/analytic code
How to access documents: Statistical/Analytic code we be available upon request via email to courtney.lee@pennmedicine.upenn.edu
When available: With publication

Additional Information
Who can access the data: Data will be made available to anyone requesting the data.
Types of analyses: Data will be made available for any purpose.
Mechanisms of data availability: Data will be available without investigator support.
